# Magnesium Level and Related Factors in Type 2 Diabetes Mellitus: A Cross-Sectional Study

**DOI:** 10.2174/0118715303411661250721141050

**Published:** 2025-08-01

**Authors:** Kamil Konur, Hatice Beyazal Polat, Erol Karavar, Teslime Ayaz

**Affiliations:** 1 Department of Internal Medicine, Faculty of Medicine, Recep Tayyip Erdoğan University, Rize, Türkiye;; 2 Department of Internal Medicine, Faculty of Medicine, Bakircay University, Izmir, Türkiye

**Keywords:** Magnesium deficiency, glycemic control, HbA1c, metformin, hypertension, Type 2 diabetes mellitus

## Abstract

**Introduction:**

Type 2 diabetes mellitus is a chronic metabolic disorder often accompanied by alterations in serum magnesium levels. This study aimed to investigate the relationship between serum magnesium concentration and glycemic control, comorbidities, and medication use in patients with type 2 diabetes mellitus.

**Methods:**

A retrospective cross-sectional analysis was conducted using data from 502 patients. Glycemic control was assessed based on HbA1c levels, and serum magnesium concentrations were evaluated concerning clinical and demographic variables. Statistical analyses included t-tests, Mann-Whitney U tests, logistic regression, and ROC curve analysis.

**Results:**

Patients with poor glycemic control had significantly lower serum magnesium levels. Magnesium levels were lower in females, particularly postmenopausal women. Magnesium levels were significantly associated with hypertension, gender, and the use of specific medications such as metformin and indapamide. Logistic regression revealed a significant inverse association between serum Magnesium levels and congestive heart failure (OR = 0.055), but not with other comorbidities. ROC analysis revealed limited predictive value of magnesium for glycemic control (AUC = 0.41).

**Discussion:**

Although group-level differences in magnesium were evident, magnesium levels alone were not reliable predictors of glycemic control. However, the associations with CHF, HT, gender, and specific medications suggest that magnesium plays a multifaceted role in type 2 diabetes mellitus management.

**Conclusion:**

Regular monitoring of serum magnesium may aid in identifying at-risk patients, especially those with hypertension, CHF, or on magnesium-depleting medications. Further prospective studies are needed to clarify the clinical utility of magnesium in diabetes care.

## 1 INTRODUCTION

Type 2 diabetes mellitus (T2DM) is a chronic metabolic disorder characterized by insulin resistance and/or deficiency, leading to persistent hyperglycemia and associated microvascular and macrovascular complications. Currently, T2DM accounts for over 90% of all diabetes cases globally, with 783 million people projected to be affected and healthcare costs expected to exceed US$1,054 billion by 2045 [1, 2]. T2DM is a complex multifactorial polygenetic disease that be attributed to many risk’ factors [3, 4]. In the 'Turkey Nutrition and Health Survey-2017', it was revealed that 13.6% of individuals aged 20 and older have diabetes. Among this age group, the Western Black Sea region reports the highest diabetes prevalence at 15.5%. Additionally, the same study found that 34% of people aged 18 and above are classified as obese. According to the STEPS-2017 study, conducted in partnership with WHO, the diabetes prevalence in those over 19 years old was noted to be 12.2%, while the rate of obesity was reported at 32% [5]. In a multicentre study conducted in Turkey with 10,121 patients, the incidence of T2DM in obese individuals was found to be 35.01% [6].

The pathophysiology of T2DM is not solely limited to insulin dynamics. Alterations in micronutrient levels also play a role in achieving glycemic control [7, 8]. In this context, recent studies have increasingly highlighted the role of magnesium (Mg) in metabolic diseases [9-11]. Mg, the second most abundant intracellular divalent cation, plays a pivotal role in numerous physiological processes, including glucose metabolism and insulin signaling. It acts as a cofactor for various enzymes involved in carbohydrate metabolism and is essential for the proper functioning of insulin receptors [12-14]. Mg deficiency is associated with pancreatic β-cell dysfunction and insulin resistance, ultimately increasing the risk of developing T2DM and metabolic syndrome. Inadequate intracellular magnesium homeostasis may enhance insulin resistance by modulating the activity of glucokinase and altering cellular glucose flux [15-17]. Hypomagnesemia, a condition characterized by low serum Mg levels, has been frequently observed in individuals with T2DM, with prevalence rates ranging from 13.5% to 47.7% [18-20].

The etiology of hypomagnesemia in T2DM is multifactorial, encompassing factors such as inadequate dietary intake, increased renal excretion, gastrointestinal losses, and the use of certain medications like metformin and diuretics [21-23]. Hyperglycemia and hyperinsulinemia increase renal Mg excretion and contribute to the development of hypomagnesemia [24]. Notably, hypomagnesemia has been linked to poor glycemic control, insulin resistance, and an increased risk of diabetic complications, including neuropathy, retinopathy, and nephropathy [25, 26].

Today, obesity and its associated comorbidities, such as metabolic syndrome and T2DM, have become major global public health concerns. At the core of obesity often lie unhealthy dietary habits characterized by high energy density and poor micronutrient content. This contributes to an increased prevalence of Mg deficiency among obese individuals [27-29]. Epidemiological data support this association. According to the National Health and Nutrition Examination Survey (NHANES III), the prevalence of Mg deficiency is significantly higher in individuals classified as obese (BMI ≥30 kg/m^2^) compared to those with normal body weight [30, 31]. Experimental studies also support these clinical findings. In diet-induced obesity models, Mg supplementation has been shown to reduce adipose tissue accumulation and improve metabolic parameters [32]. These findings suggest a potential role for Mg in the pathogenesis of obesity and highlight its metabolic effects [33-35].

Despite the established association between Mg deficiency and T2DM, routine monitoring of serum Mg levels is not commonly practiced in clinical settings. This oversight may contribute to the underdiagnosis and undertreatment of hypomagnesemia, potentially exacerbating the progression of diabetes and its complications [36].

Many methods have been used in this fight, from medical supplements to physical activity [37], from combating obesity [38-40] to surgical methods. In this research, the magnesium concentration, which is part of the medical assessments, was examined. In light of these considerations, this study aims to investigate the relationship between serum Mg levels and glycemic control in patients with T2DM. By analyzing data from a cohort of patients in Rize, Turkey, we seek to identify factors associated with Mg metabolism disturbances and assess the impact of Mg levels on glycemic outcomes. Our findings may underscore the importance of Mg monitoring in the comprehensive management of T2DM and inform strategies to mitigate the risk of diabetes related complications. Moreover, this study, based on current patient data, represents an original contribution to the literature from our region and country.

## 2 MATERIALS AND METHODS

The research was meticulously designed in compliance with the Helsinki Declaration and established ethical standards, receiving endorsement from the Ethics Committee of the Recep Tayyip Erdoğan University Faculty of Medicine on 28 November 2024, under decision number 2024/277. This investigation is characterized as a retrospective, cross-sectional analysis, with data sourced from patients who sought medical attention at the hospital in Rize between January 2023 and December 2024, extracted *via* the hospital's information management system. Institutional and ethical clearance was secured before the commencement of the investigation.

Prior to obtaining data, permission was formally requested and granted by the hospital's management of the parallel blood feud. A detailed protocol for collecting data has been developed to ensure standardised and reproducible methods for all extracted variables. According to the protocol, the inclusion criteria were patients aged 18 years or over with a confirmed diagnosis of T2DM as defined by the ICD-10 codes and a clinical evaluation. Those without a diagnosis of T2DM, those with incomplete or insufficient laboratory data, those with end-stage renal failure and undergoing dialysis treatment, those who were pregnant or breastfeeding, and those under the age of 18 were excluded from the study population.

The data was obtained in an anonymised form in order to ensure confidentiality and to comply with data protection rules. All personal identifiers have been removed before the analysis. The data included demographic characteristics (age, gender), clinical comorbidities (*e.g.*, hypertension (HT), cardiovascular disease), pharmacological treatments (including antidiabetes and antihypertensive drugs), and laboratory indicators as commonly measured. These included serum Mg, blood levels of haemoglobin A1c, and renal function parameters such as serum creatinine and glomerular filtration rate (GFR). Laboratory data were obtained from fasting blood samples analysed in the central biochemistry laboratory of the hospital using standardised automated systems and calibrated assay kits. A power analysis was performed to determine the sample size. It was found that at least 220 patient data were required for a 95% effect size. A total of 502 patients with T2DM (61,81=Mage, ±11,26)) met the inclusion criteria and were included in the final analysis.

### 2.1 Definitions

The present study identified T2DM based on the criteria defined by the American Diabetes Association (ADA) in the "Standards of Care in Diabetes—2025" guideline [41]. These criteria for diagnosis encompass a fasting plasma glucose level of ≥126 mg/dL (7.0 mmol/L), a 2-hour plasma glucose level of ≥200 mg/dL (11.1 mmol/L) during an oral glucose tolerance test, a hemoglobin A1c (HbA1c) level of ≥6.5%, or a random plasma glucose of ≥200 mg/dL in individuals exhibiting typical symptoms of hyperglycemia or experiencing a hyperglycemic crisis. Only those participants who met at least one of these diagnostic criteria, confirmed on a different day if needed, were included in the study.

Serum Mg concentrations were quantified utilizing colorimetric assay techniques on an automated biochemical analyzer situated within the hospital's central laboratory. Under established reference ranges prevalent in clinical practice, individuals exhibiting serum Mg levels ranging from 1.6 to 2.4 mg/dL were designated as normomagnesemic, whereas those with serum Mg concentrations falling below 1.6 mg/dL were identified as hypomagnesemic [42]. To assess glycaemic control, patients were stratified into two groups according to their HbA1c levels, measured by high-performance liquid chromatography (HPLC) standardized according to the national glycaemic standardization programme (NSLS). Patients with an HbA1c <7.0 percent were considered to have good glycaemic control, while patients with a value above 7.0 percent were classified as having poor glycemic control, with the ADA targets for optimal diabetes control [43]. This classification allowed a comparison of Mg status across different glycemic control profiles, which may provide insight into the metabolic impact of Mg deficiency in poorly controlled T2DM patients (61,81=Mage, ± 11,26).

### 2.2 Statistical Analysis

All statistical analyses were performed using IBM SPSS Statistics Version 26.0 (Armonk, New York, USA). Continuous variables were expressed as mean ± standard deviation (SD) or median with interquartile range (IQR), depending on the distribution assessed by the Shapiro-Wilk test. Categorical variables were presented as frequencies and percentages. Comparisons between two independent groups (*e.g.*, normomagnesemic *vs*. Hypomagnesemic; good *vs*. poor glycemic control) were performed using the Student's t-test for normally distributed variables and the Mann-Whitney U test for non-normally distributed variables. The Chi-square test (or Fisher's exact test where appropriate) was used to compare categorical variables. Correlation between continuous variables (*e.g.*, serum Mg and eGFR) was evaluated using Pearson’s or spearman’s correlation coefficients, depending on data distribution. To identify independent predictors of poor glycemic control and other comorbidities (HT, CAD, CHF, nephropathy), binary logistic regression analysis was conducted with serum magnesium level as the main independent variable. Odds ratios (OR) and 95% confidence intervals (CI) were reported. Model fit was assessed using the Chi-square statistic, Cox & Snell R^2^, and Nagelkerke R^2^ values. In addition, receiver operating characteristic (ROC) curve analysis was performed to evaluate the discriminative ability of serum magnesium levels for predicting poor glycemic control. The area under the curve (AUC) was calculated, with values closer to 1.0 indicating better discriminatory power. A two-tailed *p*-value <0.05 was considered statistically significant for all analyses.

## 3 RESULTS

**Fig. (1):**
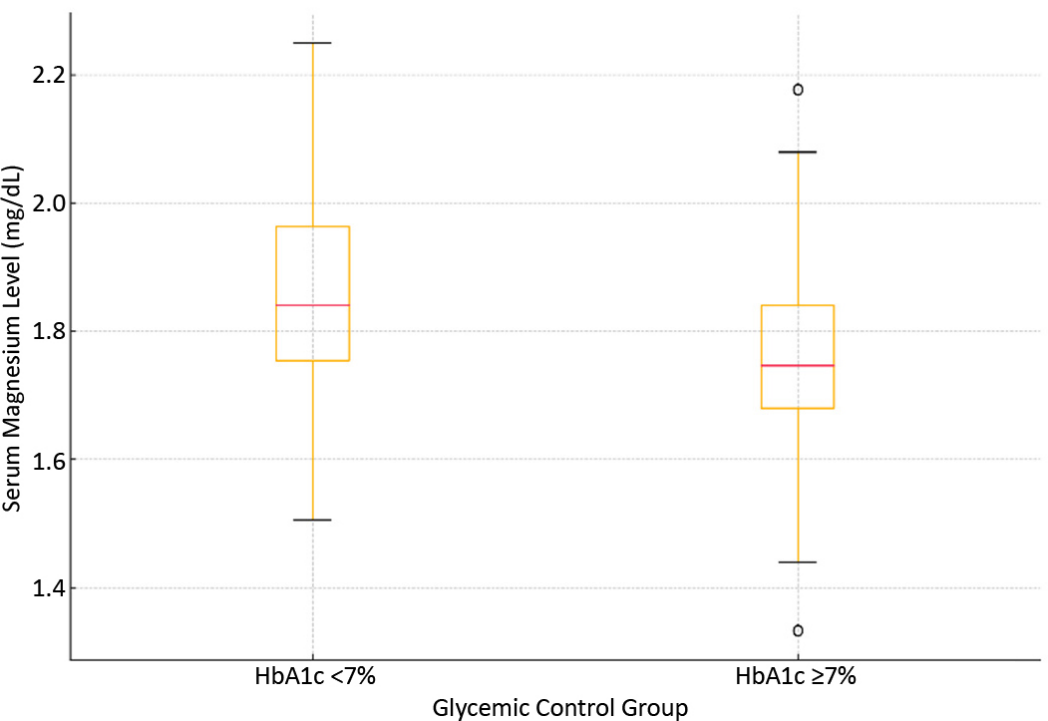
Boxplot of magnesium level by glycemic control.

The study involved a total of 502 patients, 50.4 percent female and 49.6 percent male. The mean patient age was 61.81 ± 11.26 years. Patients with comorbidities were evaluated as 79.1 percent had HT, 22.9 percent had coronary artery disease (CAD), 17.4 percent had congestive heart failure (CHF) and 58 percent had nephropathies. The mean value of the average serum HbA1c was 8.0 ± 1.8 percentage points. When the glycemic control was assessed, 31.5 percent had good glycemic control and 68.5 percent had poor glycemic control. When patients were examined for Mg, 14.3 percent had hypomagnesaemia, 84.7 percent had normomagnesaemia, and 1 percent had hypermagnesaemia. The mean Mg concentration was 1.90 mg per L and the SD was 0.22, whereas the mean Mg concentration was 1.81 mg per L in patients with poor glycemic control (SD = 0.24). When the relationship between glycemic control and Mg levels was examined, a statistically significant difference (*p* = 0.000) was found. Patients with poor glycemic control had significantly lower Mg levels (Fig. **1**).

The analyses revealed that Mg levels in diabetic patients differed significantly between genders (95% confidence interval, *p* < 0.05). The mean Mg levels in male patients with diabetes were 1.87 mg per litre (SD = 0.23), while the mean Mg levels in female patients were 1.8 mg per litre (SD = 0.23). Women were divided into two groups based on reproductive status: reproductive age and menopausal period. When comparing Mg levels between women under the age of 50 and those aged 50 or older, a statistically significant difference in mean values was observed with 95% confidence (*p*-value < 0.05). Accordingly, the mean Mg level in women under 50 was 1.91 (SD = 0.15), while it was 1.79 (SD = 0.24) in women aged 50 and above (Table **1**).

The relationship between comorbidity and serum Mg levels was found to be higher in male patients than in female patients. A statistically significant difference in Mg levels was observed between patients with and without HT (*p*-value < 0.05) when compared to placebo. The mean Mg levels in HT patients were 1.82 mg per deciliter (SD = 0.23) while the mean Mg levels in non-HT patients were 1.88 mg per deciliter (SD = 0.22). There was no significant correlation between Mg levels and other comorbidities (CAD, CHF, nephropathies) (Table **2**). There is a statistically significant positive linear correlation between GFR level and serum Mg level with 95% confidence (*p*-value < 0.05). Although the strength of the correlation is weak, it can be stated that serum Mg levels tend to decrease as GFR decreases.

The patients were divided into two groups: those with normomagnesemia (Group A) and those with hypomagnesemia (Group B), and the relationship between comorbidities and Mg levels was examined. The frequency of HT in Group A was 78.4%, while in Group B it was 83.3%; the frequency of CHF in Group A was 16.5%, while in Group B it was 18.1%; the frequency of CAD in Group A was 23.1%, while in Group B it was 20.8%; and the frequency of nephropathy in Group A was 58.6%, while in Group B it was 55.6%. No statistically significant relationship was found between the frequency of chronic diseases examined in the two groups (*p* < 0.05).

When examining the medications used by the patients, the following percentages were observed: metformin 85.3%, sodium-glucose co-transporter 2 (SGLT-2) inhibitors 51.4%, dipeptidyl peptidase-4 (DPP-4) inhibitors 45.2%, hydrochlorothiazide (HCT) 23.3%, proton pump inhibitors (PPI) 16.7%, indapamide 12.2%, pioglitazone 8.6%, and furosemide 4.8%. When comparing the medications used by the patients with their Mg levels, a statistically significant difference was found with 95% confidence in patients using metformin and indapamide compared to those not using these medications (*p*-value<0.05). The average Mg level in patients using metformin was 1.82 mg/dL (SD = 0.22), while in patients not using metformin, it was 1.91 mg/dL (SD = 0.28). Mg levels were significantly lower in patients using metformin. The average Mg level in patients using indapamide was 1.74 mg/dL (SD = 0.23), while in patients not using indapamide, it was 1.85 mg/dL (SD=0.23). Mg levels were significantly lower in patients using indapamide (Table **3**).

Binary logistic regression analyses were performed using serum magnesium level as the independent variable, and glycemic control, HT, CAD, CHF, and nephropathy as dependent variables.

**Fig. (2):**
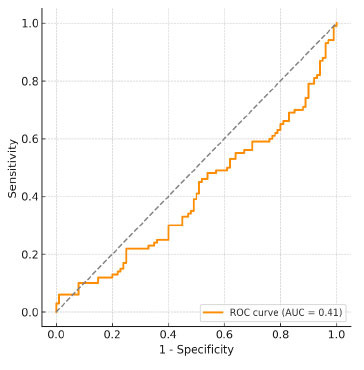
ROC Curve: Assessment of magnesium level in discriminating glycemic control.

The model established with glycemic control Chi-square=0.038, *p*<0.05, Cox & Snell R^2^=0.013, Nagelkerle R^2^=0.018. Although the chi-square value was significant, it was determined that the model parameters were not significant on their own. Despite the statistical difference in magnesium levels between patients with good and poor glycemic control, the ROC analysis and logistic regression indicate that magnesium has limited predictive power for glycemic control status (Fig. **2**).

The model for hypertension showed a Chi-square value of 0.969 (*p* > 0.05), Cox & Snell R^2^= 0.02, and Nagelkerke R^2^ = 0.03, indicating no statistical significance. Similarly, the model for coronary artery disease had a Chi-square value of 0.911 (*p* > 0.05), which was also not significant. In contrast, the model for congestive heart failure was statistically significant (Chi-square = 9.626, *p* < 0.05), with Cox & Snell R^2^= 0.019 and Nagelkerke R^2^ = 0.032. The overall classification accuracy was 83.2%. Despite the model’s limited explanatory power, it was meaningful overall, suggesting a non-random association between the variables. In this model, individuals with serum magnesium levels between 1.6 and 2.4 mg/dL had significantly lower odds of having congestive heart failure compared to those with levels below 1.6 mg/dL (OR = 0.055) (Table **4**).

## 4 DISCUSSION

In this study, the relationships between serum Mg levels and various clinical parameters in T2DM patients were examined.

When examining the relationship between gender and Mg, it was found that Mg levels were significantly lower in female patients. Considering risk factors, women in the postmenopausal period exhibited lower Mg levels compared to those in the reproductive age. Insufficient Mg intake, pregnancy and breastfeeding, menstrual cycles, and the use of birth control pills are some of the factors that may explain the lower Mg levels in females [44, 45]. A review of the literature shows studies indicating that Mg levels are lower in females [46, 47].

A reverse correlation between glycemic control and Mg levels was found. In patients with good glycemic control, Mg levels were higher, while in patients with poor glycemic control, Mg levels were significantly lower. This finding has been reported in several studies [18, 48, 49]. Mg plays a crucial role in glucose transport into cells and insulin secretion. Therefore, it is believed that mg deficiency may contribute to hyperglycemia and the complications associated with diabetes [22, 50, 51].

Although patients with poor glycemic control exhibited significantly lower serum magnesium levels in univariate analyses, the predictive value of magnesium for glycemic control was found to be limited in both the ROC analysis (AUC = 0.41) and binary logistic regression. This suggests that while a statistical difference in magnesium levels exists between glycemic control groups, serum magnesium alone may not serve as a reliable biomarker for predicting glycemic outcomes. The discrepancy may stem from the inability of magnesium levels to discriminate individual glycemic control status accurately, despite the observed group-level differences.

The significantly lower Mg levels in T2DM patients with HT highlight the important role of Mg in blood pressure regulation. Mg contributes to the regulation of vascular tone and the improvement of endothelial function, thereby protecting against the harmful effects of hypertension [52-54]. These protective effects may be diminished in diabetic and hypertensive patients with low magnesium levels. On the contrary, Mg deficiency may lead to endothelial dysfunction, further impairing vascular health and rendering endothelial cells more vulnerable to the damaging effects of oxidative stress. A positive association between decreased Mg levels and the risk of developing hypertension has been previously demonstrated [55-57]. Some studies have shown that Mg supplementation may modestly reduce blood pressure levels [58, 59]. However, further studies are needed to better elucidate the relationship between elevated blood pressure stages, uncontrolled hypertension, and serum Mg concentrations.

Several studies have shown that low serum Mg concentrations or inadequate Mg intake are associated with CHF [60-63]. Another observational study reported an inverse relationship between serum Mg levels and the risk of developing CHF, atrial fibrillation, and microvascular complications in individuals with T2DM [64]. In our study, while the univariate comparison between patients with and without CHF did not reveal a statistically significant difference in serum magnesium levels, the binary logistic regression analysis demonstrated a significant association. Specifically, patients with serum magnesium levels between 1.6-2.4 mg/dL had significantly lower odds of having CHF compared to those with levels below 1.6 mg/dL. This discrepancy may be attributed to the greater sensitivity of regression models in detecting independent predictive effects after adjusting for potential confounders. In contrast to simple group comparisons, logistic regression allows for the identification of underlying associations that may not be immediately apparent in bivariate analyses.

The significantly lower Mg levels in patients using metformin may be due to the development of losses associated with long-term use of metformin, in addition to diabetes related gastroparesis and autonomic dysfunction, as well as gastrointestinal side effects. There are studies in the literature supporting this finding [65, 66]. Indapamide is a thiazide-like diuretic agent used in antihypertensive therapy. Chronic use of thiazide class drugs has been shown to decrease serum Mg levels through renal excretion [67]. A review of the literature reveals studies indicating that indapamide use does not significantly affect Mg levels [68, 69]. In contrast to these findings, our study concluded that indapamide use significantly decreased Mg levels. This may be due to the contribution of other factors that lower Mg levels in T2DM patients.

There are studies in the literature investigating the effects of SGLT-2 inhibitors on Mg levels [70-73]. In a meta-analysis that included 18 randomized controlled trials with 15,309 patients, it was found that patients treated with SGLT-2 inhibitors had a statistically significant increase in Mg levels compared to the placebo control group [74]. In our study, no significant relationship was found between the use of SGLT-2 inhibitors and Mg levels (*p* = 0.261).

We examined the relationships between Mg levels and comorbidities in T2DM patients. In patients with HT, Mg levels were found to be significantly lower. Studies in clinical and animal research have shown that low magnesium levels increase blood pressure. Additionally, a study conducted on 4,272 patients found that low magnesium levels were significantly associated with an increased prevalence of prehypertension [75]. In this regard, our results were consistent with the literature. In our study, no significant relationship was found between diabetic nephropathy and Mg levels. Other studies have shown that low Mg levels may play a role in the development and progression of nephropathy [76]. We identified a correlation between magnesium levels and GFR. Previous studies have reported that lower magnesium levels are associated with poorer renal outcomes in patients diagnosed with chronic kidney disease [77].

In light of all these findings, regular monitoring of serum Mg levels in patients with T2DM may be beneficial. Comorbid conditions and medications that may influence Mg status should be carefully considered. Mg replacement should be considered in appropriate patients. Given the continuous rise in metabolic disorders, particularly diabetes, we believe that the clinical utility of Mg should be further emphasized.

Our study has some limitations. It has a retrospective cross-sectional design, and Mg levels were assessed with only a single measurement. A longer follow-up of patients over a specific period may yield better results. The duration of diabetes diagnosis in the patients and the duration of medication use are not known. Additionally, due to the lack of available data, associations with body mass index, obesity, other comorbid conditions, and additional diabetic complications could not be examined. Although we investigated the relationship between serum magnesium and heart failure, potential confounding variables that could have influenced the results were not included in the study. Another important limitation was the lack of information regarding whether patients were receiving Mg replacement therapy at the time of evaluation.

## CONCLUSION

Serum Mg levels in patients with T2DM are significantly associated with the presence of HT, gender, and the use of certain medications. These findings highlight the importance of monitoring Mg levels in T2DM management and suggest that strategies to address Mg deficiency could be considered, particularly in patients receiving HT treatment and metformin therapy. In the future, prospective and larger-scale studies will provide more detailed insights into the effects of Mg deficiency in T2DM, guiding treatment approaches.

## Figures and Tables

**Table 1:** Mg levels in women during the reproductive age and postmenopausal period.

Mg*Age	**Mean**	**SD**	**Median**	**Test**	***P* Value**
**Under 50**	1.91	0.15	1.95	t-testi	0.020*
**50 and over**	1.79	0.24	1.8		

**Note: ** *Since *p*-value<0.05, there is a significant difference with 95% confidence.

**Table 2:** The relationship between comorbidities and serum Mg levels.

**Variables**	**n**	**Mg** **x̄ ± Ss**	**Statistical Analysis** Test (Mann Whitney)/*p*-value
HT	-	-	-
No	105	1.88 ± 0.22	-2.116/0.034*
Yes	397	1.82 ± 0.23	-
CHF	-	-	-
No	414	1.84 ± 0.23	-0.759/0.448
Yes	87	1.83 ± 0.26	-
CAD	-	-	-
No	387	1.83 ± 0.23	-0.078/0.938
Yes	115	1.84 ± 0.23	-
NEPHROPATHY	-	-	-
No	211	1.83 ± 0.24	-0.166/0.868
Yes	291	1.84 ± 0.23	-

**Note: ** *Since *p*-value<0.05, there is a significant difference with 95% confidence.

**Table 3:** The relationship between gender, medication, and serum magnesium level.

**Variables**	**n**	**Mg** **x̄ ± Ss**	**Statistical Analysis** Test (Mann Whitney)/*p*-value
GENDER	-	-	-
Female	253	1.8 ± 0.23	-2.54/0.012*
Male	249	1.87 ± 0.23	-
METFORMIN	-	-	-
Not use	74	1.91 ± 0.28	-2.096/0.036*
Use	428	1.82 ± 0.22	-
DPP-4	-	-	-
Not use	275	1.84 ± 0.23	-1.268/0.205
Use	227	1.82 ± 0.23	-
SGLT-2	-	-	-
Not use	244	1.82 ± 0.23	-1.123/0.261
Use	258	1.85 ± 0.23	-
PIOGLITAZON	-	-	-
Not use	459	1.83 ± 0.23	-0.953/0.34
Use	43	1.86 ± 0.25	-
FUROSEMID	-	-	-
Not use	478	1.84 ± 0.23	-1.007/0.314
Use	24	1.81 ± 0.31	-
HCT	-	-	-
Not use	385	1.85 ± 0.23	-2.053/0.4
Use	117	1.79 ± 0.24	-
PPI	-	-	-
Not use	418	1.84 ± 0.23	-0.196/0.844
Use	84	1.83 ± 0.24	-
INDAPAMID	-	-	-
Not use	441	1.85 ± 0.23	-3,224/0,001*
Use	61	1.74 ± 0.23	-

**Note: ** *Since *p*-value<0.05, there is a significant difference with 95% confidence.

**Table 4:** Binary logistic regression analysis of serum magnesium levels and clinical outcomes.

-	B	S.E.	Wald	Sig.	Exp(B) (Odds Ratio)
Glycemic Control	-	-	-	-	-
Mg < 1.6	-	-	5.868	0.053	-
Mg = 1.6 - 2.4	0.035	1.157	0.001	0.976	1.036
HT	-	-	-	-	-
Mg < 1.6	-	-	0.920	0.631	-
Mg = 1.6 - 2.4	0,223	1.162	0.037	0.848	1.250
CAD	-	-	-	-	-
Mg < 1.6	-	-	0.964	0.618	-
Mg = 1.6 - 2.4	-0,930	0.958	0.942	0.332	0.395
CHF	-	-	-	-	-
Mg < 1.6	-	-	7.178	0,028*	-
Mg = 1.6 - 2.4	-2.899	1.159	6.253	0.012*	0.055
Nephropathy	-	-	-	-	-
Mg < 1.6	-	-	0.873	0.646	-
Mg = 1.6 - 2.4	0,629	0.943	0.444	0.505	1.875

**Note: ** *Since *p*-value<0.05, there is a significant difference with 95% confidence.

## Data Availability

All data generated or analyzed during this study are included in this published article.

## LIST OF ABBREVIATIONS

T2DM: Type 2 diabetes mellitus
WHO: World Health Organization
Mg: Magnesium
BMI: Body mass index
HT: Hypertension
CHF: Congestive heart failure
CAD: Coronary artery disease
GFR: Glomerular filtration rate
SD: Standard deviation

## STANDARDS OF REPORTING

STROBE guidelines were followed.
